# Intelligence and Disability Pension in Swedish Men and Women Followed from Childhood to Late Middle Age

**DOI:** 10.1371/journal.pone.0128834

**Published:** 2015-06-10

**Authors:** Andreas Lundin, Alma Sörberg Wallin, Daniel Falkstedt, Peter Allebeck, Tomas Hemmingsson

**Affiliations:** 1 Institute of Environmental Medicine, Karolinska Institutet, Stockholm, Sweden; 2 Department of Public Health Sciences, Karolinska Institutet, Stockholm, Sweden; 3 Centre for Social Research on Alcohol and Drugs, Stockholm University, Stockholm, Sweden; Örebro University, SWEDEN

## Abstract

**Objective:**

To investigate the association between intelligence and disability pension due to mental, musculoskeletal, cardiovascular, and substance-use disorders among men and women, and to assess the role of childhood social factors and adulthood work characteristics.

**Methods:**

Two random samples of men and women born 1948 and 1953 (n = 10 563 and 9 434), and tested for general intelligence at age 13, were followed in registers for disability pension until 2009. Physical and psychological strains in adulthood were assessed using job exposure matrices. Associations were examined using Cox proportional hazard regression models, with increases in rates reported as hazard ratios (HRs) with 95% confidence intervals (95%CI) per decrease in stanine intelligence.

**Results:**

In both men and women increased risks were found for disability pension due to all causes, musculoskeletal disorder, mental disorder other than substance use, and cardiovascular disease as intelligence decreased. Increased risk was also found for substance use disorder in men. In multivariate models, HRs were attenuated after controlling for pre-school plans in adolescence, and low job control and high physical strain in adulthood. In the fully adjusted model, increased HRs remained for all causes (male HR 1.11, 95%CI 1.07–1.15, female HR 1.06, 95%CI 1.02–1.09) and musculoskeletal disorder (male HR 1.16, 95%CI 1.09–1.24, female HR 1.08, 95%CI 1.03–1.14) during 1986 to 2009.

**Conclusion:**

Relatively low childhood intelligence is associated with increased risk of disability pension due to musculoskeletal disorder in both men and women, even after adjustment for risk factors for disability pension measured over the life course.

## Introduction

Disability pension, i. e., economic compensation when a person's work ability becomes chronically reduced due to illness, injury or disability, is one of the cornerstones of the Swedish social security system, and is second only to old age pension in terms of expenditure. In most cases, receipt of disability pension entails permanently leaving the labor market. In 2013, about 7% of 19–64 year-olds had fully or partially left the labor market with a disability pension [1]. Although the pension decision is based on severity of functional impairment with regard to work, as judged by a physician, the granting of compensation is made with reference to the illness, injury or disability as it is registered in the International Classification of Diseases (ICD). Mental and musculoskeletal disorders are the dominant diagnostic groups [1]. Previously identified risk factors for disability pension are low education [2,3], current mentally and physically strenuous working conditions [2,4,5] low employment status [2], poor health behaviors, such as smoking, high alcohol consumption and lack of exercise, and body composition [2,4,6].

Intelligence has also been linked to health, and health-related and care-seeking behaviors, with risks increasing as intelligence decreases [7]. People with lower pre-morbid intelligence are more likely to smoke [8,9,10] and drink excessively [11,12], and have a higher non-adherence to treatment [13,14]. Furthermore, intelligence is, in most societies, the primary determinant of educational attainment, which in turn determines whether occupational positions and working conditions entail more or less physical and psychological strain [15]. A few studies, all of which are based on conscripted men, have established an association between level of intelligence and disability pension [16,17,18], but only one has made use of information on cause-specific disability pension [17]. A large study of Norwegian conscripts has shown that intelligence predicts disability pension in early adulthood (ages 23–36) [16], whereas studies based on Swedish conscripts have found that intelligence predicts disability pension at both ages 20–43 [17] and 40–59 [18]. In one study of early-life predictors of disability pension in conscripts, which focused specifically on psychiatric diagnoses, people with low intelligence (defined as members of stanine groups 1–4, i.e., the 40% of the population lowest in intelligence) were found to have increased risks of disability pension due to psychosis, non-psychotic disorder, alcohol-use disorder and drug-related disorder, compared with those with medium to high intelligence [17]. We have, in longer follow-up of the same conscripts, although not those with cause-specific diagnoses, examined the potential mediating role of working conditions, showing that adult working conditions mediate part of the association between young-adulthood intelligence and middle-age disability pension [18]. The same measures of working conditions have also been shown to explain educational differences in disability pension [5].

No previous study has examined the association between intelligence and disability pension in women, which may be different from that demonstrated in men. Moreover, no study has made use of disability pension diagnosis, which offers more specific information on the morbidity behind the functional impairment that may associate intelligence with disability pension in middle age. Intelligence may be an integral part in the production of social health inequalities, through factors such as behavior, and labor-market and working conditions (factors which we also know may differ according to gender). Time series studies has also shown that the expansion of the education system potentially lead to increased wage inequality [19], and those with a higher education are less likely to have longer unemployment spells [20]. Intelligence is a strong predictor of education, and a very large study has recently shown that education has remained a strong mediator in the association between male intelligence and all-cause disability pension across two decades [21]. However, it is also possible that intelligence is a risk factor for disability pension independent of mechanisms associated with education.

In the present study, we aim to investigate the associations between intelligence and disability pension, all-cause and cause-specific, in two pooled samples of 13-year-old boys and girls born in 1948 and 1953 and followed in national registers up to 2009. While all-cause disability pension is a summary measure of the extent of functional disability and exclusion from the labor market, cause-specific disorders may provide information regarding the mechanisms involved. That is, people are excluded from the labor market for not meeting mental or cognitive demands, for musculoskeletal disorder arising from manual work, or for decreased work ability due to life-style-related diseases, and the specific morbidity behind the disability pension may contribute to explain whether intelligence is a risk factor for not meeting physical, mental or other functional demands. Because these categories are not clear-cut–cardiovascular disease, for example, is caused not only by life-style related factors but also by work-related stress–an additional objective is to investigate the extent to which adult working conditions, measured using psychosocial and physical Job Exposure Matrices (JEMs), mediate any associations between intelligence and disability pension due to mental disorder, substance use disorder, musculoskeletal disorder, and cardiovascular disease (CVD).

## Method and Variables

### Ethics statement

Ethical approval was obtained from Stockholm’s Regional Ethical Review Board at Karolinska Institutet (decision reference number 2004/5:9–639/5). Due to the character of the database and the anonymization of all data, the Review Board waived the normal requirement of written consent.

### Study population

**Fig 1:**
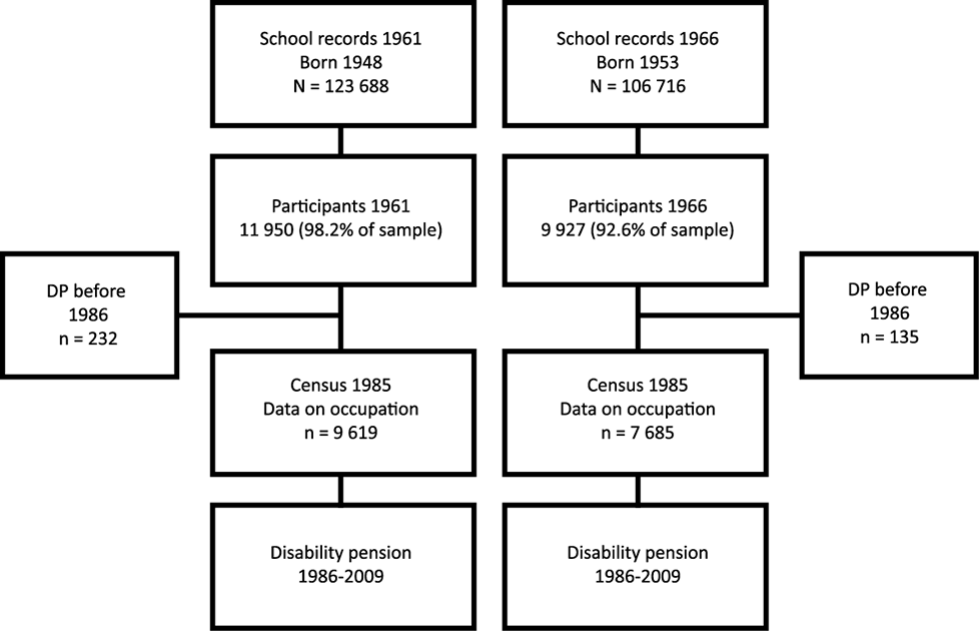
Study Components of the Evaluation Through Follow-up 1948 and 1953 cohorts. The figure shows the sample frame, initial participants, deaths between participation and the 1986 census, and the study base in which disability pension was studied.

We used data from the 1961 and 1966 waves of the cohort-sequential longitudinal database Evaluation Through Follow-up (Swedish acronym UGU) which comprises nationally representative samples of people born 1948 and 1953, and are collected for the purposes of national school evaluation programs and social science research. Both cohorts were sampled in the spring, and the sample frame consisted of individuals born on the 5^th^, 15^th^ and 25^th^ of each month during the target years 1948 and 1953 (Originally 1948 n = 12 166 and 1953 n = 10 723, in our sample 1948 n = 11 988, of which 5818 were girls, and 1953 n = 9881 of which 4891 were girls). Intelligence tests and questionnaires were administered in school, usually to 6^th^ graders (age 13), and completed by 10 562 individuals in 1961 and 9 434 in 1966. The data were administered by the Department of Education and Educational Research at Gothenburg University in cooperation with Statistics Sweden [22]. The participation rate was 98.2% in 1961, and 92.6% in 1966, the difference being due to a longer examination period in 1961 [22]. Analysis of non-participation showed no differences in sex or social class of origin between participants and non-participants [23]. The two cohorts were pooled and linked to information from administrative records on cause and date of disability pension, and also to the Swedish census of 1985. An overview of the data is presented in Fig 1. Because disability pension is part of the national basic pension and insurance system, which covers all Swedish residents, everyone in a cohort is considered at risk, irrespective of labor-market status.

### The age-13 intelligence test

*Intelligence test results at 13* consist in the sum of scores from three tests: verbal (antonyms, 40 items, 10 minutes), inductive (number series, 40 items, 18 minutes), and spatial (metal folding, 40 items, 15 minutes). The tests measure the verbal, inductive and spatial aspects of intelligence separately, developed according to a Thurstone classification of abilities, but it has been shown that they can be combined as a measure of general intelligence (*g*) [24].

The tests were administered by class teachers according to instructions handed over by the school head teacher. They were performed in two sessions with a break in-between. Session 1 started with antonyms, followed by metal folding, and then a questionnaire on leisure. Session 2 started with a questionnaire on future plans, followed by the number series test, and questionnaires on interests and attitudes toward school. All tests started out with easy items but became progressively more difficult, and teachers were instructed to help with the written instructions. All tests were preceded by examples illustrated by the teachers. Reliability tests were conducted on a subsample (173 boys and 176 girls, born 1948), showing that intercorrelations ranged between 0.41 and 0.48, and that internal consistency was good (Kuder–Richardson Formula 20 coefficients: 0.87 for the verbal, 0.88 for the spatial, and 0.92 for the inductive test) [25]. The tests developed for these cohorts are the same that were later used for the Swedish and Danish Metropolit cohorts of 1953 [26,27]. For each individual, we summed the scores of the three tests, and standardized the total into a stanine score as a measure of general intelligence.

### Disability pension

Information on disability pension is registered yearly in the Swedish Social Insurance Agency’s database. Anyone between 16 and 65 years of age can be granted disability pension if their working capacity is impaired by at least 25% (50% prior to 1993) [28]. The following diagnostic groups were used: mental disorder excluding substance use ICD-8 290–302, 306–311, ICD-9 290–302, 306–315, and ICD-10 F00–F09, F20–F99; substance use disorder; ICD-8 and ICD-9 303–305, ICD-10 F10–F19; musculoskeletal diagnosis ICD-8 710–738, ICD-9 710–739 and ICD-10 M00–M99; and; cardiovascular disease ICD-8 390–459, ICD-9 390–459, ICD-10 I00–I99.

### Covariates

#### Work characteristics in adulthood

High psychological demands/Low job control and High physical strain at work were established using two Job Exposure Matrices linked to the individuals’ 5-digit occupational codes, as registered in the National Population and Housing Census of 1985 (Nordic Classification of Occupations, NYK, the Swedish version of ISCO-88). The Psychosocial Job Exposure Matrix is based on questions from the Swedish Work Environment Surveys 1989–1997 concerning different aspects of working conditions, e.g., psychological demands (five items) and job control (four items on decision authority, and three on skill discretion). Average scores on these factors for 321 job titles were ascribed to the participants [29]. We created four equally sized groups based on psychological demands and job control: high, medium–high, medium–low, and low. In the manual the spearman-rank-order correlation between the derived JEM group and individual exposure level group for psychological demands ranged between 0.25 and 0.31 in men and women age 30–44, and for job control between 0.39 and 0.45 [29]. The second matrix, Physical Strain at Work, is based on a summary index of eight physical risk factors in the Annual Level-of-Living Surveys (1977, 1979–1981): heavy lifts daily, repetitive and one-sided work movements, awkward work postures, heavy shaking or vibrations, daily perspiration from physical exertion, contact with dirt, deafening noise, and risk of exposure to accidents, with means calculated for 300 job titles. Means were grouped into quartiles of physical strain, labeled high, medium–high, medium–low, and low [30], which we ascribed to the participants. We examined the spearman-rank-order correlation between the derived JEM group and the individual exposure level (a linear combination of the dichotomously scored items of physical work environment) in the Annual Level-of-Living Survey 1977 linked to the JEM by NYK (with information on both: n = 7282) and found it to be 0.51 for women and 0.52 for men. Both matrices have been used previously as proxies for mental and physical working conditions [18].

#### Level of education

Data on level of education were taken from administrative school records and registered according to the Swedish version of the International Standard Classification of Education (ISCED): 1) Primary and lower secondary, < 9 years; 2) Primary and lower secondary, 9 years; 3) Secondary; 4) Upper secondary; 5) Post-secondary, 2 years or less; 6) Post-secondary, 3–4 years, and; 7) Postgraduate education. We combined categories 1 and 2 (≤9 years of schooling), and 6–7 (≥15 years of schooling) leaving five groups based on number of years of education: ≤9, 10–11, 12–13, 14, and ≥15 years. Very few persons were found in groups 1 and 7 in this cohort.

#### School plans and paternal education

Because intelligence test might also reflect differences in school motivation, or childhood circumstances we included two measures of potential confounders, future school plans and paternal education. School plans, the intent to study beyond compulsory schooling, was measured using the item: ‘Do you expect eventually to graduate from post-secondary school?’/’Will you eventually apply for post-secondary school?’ with response options ‘Yes’, ‘No’, and ‘Don’t know’. The rationale for using this item was that it has been shown to be a good predictor of future school continuation [23]. The highest level of education of the father (or other male caretaker) was reported in a questionnaire with four pre-defined categories: primary (compulsory six years), lower secondary (nine years), upper secondary (twelve years) and higher education (more than twelve years). Low education (in descriptive tables) was defined as primary education only. A total of 6.5% and 9.5% gave no information on paternal education and school plans, respectively.

### Statistical analysis

The association between intelligence and disability pension was examined using Cox proportional hazards regression modeling, treating intelligence as a continuous variable, in order to measure increases in risk with decreasing levels of intelligence. Crude as well as adjusted hazard ratios (HRs) are reported, and 95% confidence intervals (CIs) are used as measures of uncertainty. Person-years were counted from January 1, 1971 until the date of receiving disability pension, migrating, dying or until end of follow-up on July 1, 2009. Non-proportionality was tested by including interaction by time in the regressions. School plans and paternal education were included as potential confounders, together with control for cohort (individuals born 1948 or 1953). In the analyses examining the potential mediating factors i.e. the role of education, information on which was first available in 1986, and the occupational factors, which were attributed from the occupational titles measured in 1985, person-years were counted from January 1, 1986. To assess the role of working conditions, we conducted the same analyses, but restricted the sample to people with occupational titles measured in 1985. After controlling for confounders and education, the potential mediators, i.e., physical demands, psychological demands and work control were added to the model. As a last step, we excluded education from the full analysis, since there are debates over whether education represents intelligence, whether it is a proxy for occupational exposures, and whether it represents additional, independent factors, such as personality or incentives [15]. We used listwise deletion, and all analyses were conducted on 18 535 individuals (85% of the sample). Regressions were performed in SAS using the PHREG procedure (SAS 9.3, SAS Institute Inc., Cary, NC, USA).

## Results

Of the total study population, 17.6% (n = 3269) had been granted a disability pension by the end of follow-up in 2009, by sex, 13.3% of the men (n = 1238) and 22.1% of the women (n = 2031).

Table 1 shows the distribution of potential explanatory risk factors across groups based on intelligence (with stanines 1–3, 4–6 and 7–9 grouped for illustrative purposes. Only individuals with full information on all variables are included). Individuals with higher intelligence more often came from homes with educated fathers, and more often thought they would continue into secondary school. Work characteristics of the jobs reported by the individuals in 1985, at ages 32–37, also differed when they were grouped by intelligence; physically demanding jobs and low-control jobs were more common among those with lower intelligence, as too were jobs with lower psychological demands.

**Table 1:** Distribution of potential explanatory risk factors across groups based on intelligence.

	All			Intelligence	
			Low	Middle	High
			(Stanines 1–3)	(Stanines 4–6)	(Stanines 7–9)
*Men*	*n* = 9 345		*n* = 2 021	*n* = 4 920	*n* = 2 203
	n	%	%	%	%
Father education, low	7571	81.0	90.8	82.6	69.6
School plans, no	3569	37.9	59.1	38.9	18.6
Education, ≤9 years^a^	2679	32.6	54.2	32.8	14.7
Physical demands, high^a^	2180	22.5	40.5	29.4	16.8
Psychological demands, high^a^	2340	26.5	11.9	24.9	41.8
Work control, low^a^	1632	19.8	33.3	19.0	10.7
*Women*		*n* = 9 190	*n* = 2 125	*n* = 5 119	*n* = 1 946
	n	%	%	%	%
Father’s education, low	7464	81.2	90.0	82.2	69.1
School plans, no	3198	34.6	52.4	34.9	15.2
Education, ≤9 years ^a^	1821	25.3	42.9	24.1	9.9
Physical demands, high^a^	755	37.1	19.3	9.3	4.3
Psychological demands, high^a^	1765	24.4	10.0	24.1	40.5
Work control, low^a^	2220	30.9	47.5	29.5	16.9

^*a*^ = Participants with information on occupation in 1985 census (n = 8218 men, 7213 women).

Table 2 shows the associations between potential explanatory risk factors and disability pension 1971–2009 as hazard ratios, adjusted for cohort. With the exception of the father being lowly educated, which was not a predictor in men, all the risk factors were statistically significant predictors of all-cause disability pension. Low psychological demands were associated with increased risk of disability pension.

**Table 2:** Associations between potential explanatory risk factors and disability pension, hazard ratios (HRs) and 95% confidence intervals (CIs).

		Men	Women
		HR	HR
		[95% CI]	[95% CI]
*Father’s education*	Low	1.11	1.20
		[0.92–1.35]	[1.03–1.37]
	Not low	1	1
*School plans*	Don’t know	1.09	1.21
		[0.88–1.34]	[1.04–1.42]
	No	1.35	1.49
		[1.09–1.66]	[1.27–1.75]
	Continue	1	1
Education ^*a*^	≤ 9 years	2.86	1.62
		[2.15–3.82]	[1.34–1.96]
	10–11 years	2.44	1.22
		[1.82–3.25]	[1.02–1.48]
	12–13 years	2.03	0.87
		[1.49–2.77]	[0.67–1.13]
	14 years	1.45	0.93
		[0.99–2.11]	[0.75–1.17]
	≥15 years	1	1
*Physical demands* ^*a*^	High	1.62	2.10
		[1.32–1.99]	[1.77–2.49]
	Medium-high	1.64	1.58
		[1.32–2.06]	[1.37–1.83]
	Medium-low	1.06	1.08
		[0.85–1.33]	[0.93–1.25]
	Low	1	1
*Psychological demands* ^*a*^	High	0.53	0.50
		[0.43–0.65]	[0.42–0.59]
	Medium-high	0.64	0.62
		[0.53–0.76]	[0.53–0.73]
	Medium-low	0.81	0.68
		[0.68–0.98]	[0.59–0.78]
	Low	1	1
*Work control* ^*a*^	Low	1.78	2.09
		[1.47–2.15]	[1.64–2.66]
	Medium-low	1.59	1.42
		[1.30–1.95]	[1.12–1.82]
	Medium-high	1.40	1.53
		[1.16–1.69]	[1.19–1.96]
	High	1	1

^*a*^ = Participants with information on occupation in 1985 census (n = 8218 men, 7213 women).

Table 3 shows the associations between intelligence and disability pension from all causes, mental disorder, substance use disorder, musculoskeletal disorder and cardiovascular disease starting from 1971 (*n* = 9345 men and *n* = 9190 women), and also from 1986 (*n* = 9230 men and *n* = 9037 women). With the exception of the small group of women with disability pension granted for substance use disorder, all associations remained significant after controlling for childhood circumstances in both the longer follow-up starting in 1971 and in the follow-up starting in 1986. After starting follow-up in 1986, and adjusting in the analyses for achieved education, only disability from all causes and from musculoskeletal disorder remained significantly statistically associated with intelligence.

**Table 3:** Intelligence and relative risks of disability pension 1971–2009 and 1986–2009, hazard ratios (HRs) and 95% confidence intervals (CIs) associated with one point decrease on the stanine intelligence scale.

	Men					Women				
Stanine (per step decrease)	All-cause n = 1238	Psychiatric non substance n = 371	Substance n = 77	Musculo-skeletal n = 376	CVD n = 99	All-cause n = 2031	Psychiatric nonsubstance n = 579	Substance n = 21	Musculo-skeletal n = 831	CVD n = 72
	HR	HR	HR	HR	HR	HR	HR	HR	HR	HR
	[95% CI]	[95% CI]	[95% CI]	[95% CI]	[95% CI]	[95% CI]	[95% CI]	[95% CI]	[95% CI]	[95% CI]
Model 1^a^	1.20	1.13	1.25	1.30	1.16	1.15	1.08	1.17	1.23	1.16
	[1.17–1.24]	[1.07–1.20]	[1.11–1.40]	[1.23–1.37]	[1.05–1.28]	[1.13–1.18]	[1.04–1.13]	[0.93–1.47]	[1.19–1.28]	[1.02–1.314]
Model 2^b^	1.18	1.15	1.22	1.23	1.13	1.12	1.09	1.13	1.16	1.15
	[1.14–1.22]	[1.08–1.22]	[1.07–1.38]	[1.17–1.31]	[1.01–1.25]	[1.09–1.15]	[1.04–1.14]	[0.88–1.44]	[1.11–1.21]	[1.00–1.31]
	All n = 1124	Psychiatric non substance n = 241	Substance n = 66	Musculo-skeletal n = 367	CVD n = 99	All n = 1878	Psychiatric non substance n = 493	Substance n = 16	Musculo- skeletal n = 802	CVD n = 66
	HR	HR	HR	HR	HR	HR	HR	HR	HR	HR
	[95% CI]	[95% CI]	[95% CI]	[95% CI]	[95% CI]	[95% CI]	[95% CI]	[95% CI]	[95% CI]	[95% CI]
*Alive 1986*										
Model 1^a^	1.20	1.08	1.22	1.29	1.16	1.15	1.07	1.15	1.22	1.15
	[1.16–1.23]	[1.01–1.15]	[1.08–1.38]	[1.22–1.36]	[1.05–1.28]	[1.12–1.17]	[1.02–1.12]	[0.88–1.50]	[1.18–1.27]	[1.01–1.31]
Model 2^b^	1.17	1.09	1.19	1.22	1.13	1.11	1.07	1.13	1.15	1.13
	[1.13–1.21]	[1.01–1.17]	[1.04–1.36]	[1.15–1.29]	[1.01–1.25]	[1.08–1.14]	[1.02–1.13]	[0.85–1.50]	[1.11–1.20]	[0.98–1.30]
Model 3^c^	1.12	1.06	1.11	1.15	1.07	1.08	1.02	1.10	1.11	1.12
	[1.08–1.16]	[0.98–1.16]	[0.91–1.35]	[1.09–1.22]	[0.95–1.21]	[1.05–1.11]	[0.96–1.09]	[0.70–1.73]	[1.06–1.15]	[0.96–1.31]

^a^Adjusted for cohort.

^b^Adjusted for cohort, father’s education, and school plans.

^c^Adjusted for cohort, father’s education, school plans, and education.

Table 4 shows the association between intelligence and disability pension 1986–2009 among people with census information on occupation in 1985 (*n* = 8218 men and *n* = 7210 women). The strong associations of intelligence with musculoskeletal disorder and all-cause disability pension, for both men and women, remained significant after controlling for potential confounders, including education. There was no effect of restricting the analysis to those with information on occupation (cf. Tables 4 and 3). For both men and women, the confounder-adjusted HR between intelligence and disability pension granted for musculoskeletal disorder is additionally diluted by controlling for working conditions, but remains statistically significantly increased. Also, the association between intelligence and all-cause disability pension remains statistically significant in the fully adjusted model.

**Table 4:** Intelligence and relative risks of disability pension 1986–2009 in the working population, hazard ratios (HRs) and 95% confidence intervals (CI) associated with a one point decrease on the stanine intelligence scale.

	Men					Women				
Stanine (per step decrease)	All-cause n = 889	Psychiatric non substance n = 173	Substance n = 31	Musculo- skeletal n = 307	CVD n = 89	All-cause n = 1416	Psychiatric non substance n = 364	Substance n = 7	Musculo- skeletal n = 617	CVD n = 56
*Alive with occupation 1985*	HR	HR	HR	HR	HR	HR	HR	HR	HR	HR
	[95% CI]	[95% CI]	[95% CI]	[95% CI]	[95% CI]	[95% CI]	[95% CI]	[95% CI]	[95% CI]	[95% CI]
Model 1^a^	1.20	1.07	1.31	1.32	1.13	1.14	1.03	1.14	1.23	1.14
	[1.16–1.24]	[1.00–1.16]	[1.09–1.57]	[1.24–1.40]	[1.02–1.25]	[1.11–1.17]	[0.98–1.09]	[0.76–1.70]	[1.18–1.29]	[0.99–1.31]
Model 2^b^	1.17	1.09	1.21	1.25	1.10	1.10	1.03	1.14	1.16	1.11
	[1.13–1.21]	[1.01–1.18]	[1.00–1.48]	[1.18–1.33]	[0.98–1.23]	[1.07–1.14]	[0.97–1.09]	[0.74–1.77]	[1.11–1.22]	[0.96–1.30]
Model 3^c^	1.12	1.06	1.11	1.19	1.07	1.08	1.02	1.10	1.12	1.12
	[1.08–1.17]	[0.98–1.16]	[0.91–1.36]	[1.11–1.27]	[0.95–1.21]	[1.05–1.11]	[0.96–1.09]	[0.70–1.73]	[1.07–1.18]	[0.96–1.31]
Model 4^d^	1.12	1.06	1.10	1.17	1.07	1.06	1.02	1.08	1.09	1.14
	[1.08–1.16]	[0.98–1.16]	[0.90–1.35]	[1.10–1.25]	[0.95–1.20]	[1.03–1.10]	[0.95–1.08]	[0.68–1.67]	[1.04–1.14]	[0.97–1.34]
Model 5^e^	1.11	1.05	1.07	1.16	1.05	1.06	1.01	1.09	1.09	1.12
	[1.07–1.15]	[0.96–1.15]	[0.87–1.31]	[1.09–1.24]	[0.94–1.19]	[1.03–1.10]	[0.95–1.08]	[0.68–1.74]	[1.04–1.15]	[0.95–1.31]
Model 6^f^	1.11	1.05	1.07	1.16	1.06	1.06	1.01	1.08	1.08	1.13
	[1.07–1.15]	[0.96–1.15]	[0.87–1.31]	[1.09–1.24]	[0.94–1.19]	[1.02–1.09]	[0.95–1.08]	[0.68–1.72]	[1.03–1.14]	[0.96–1.33]
Model 7^g^	1.13	1.07	1.13	1.19	1.07	1.06	1.01	1.06	1.10	1.12
	[1.09–1.17]	[0.98–1.16]	[0.92–1.38]	[1.11–1.26]	[0.95–1.20]	[1.03–1.10]	[0.95–1.07]	[0.68–1.67]	[1.05–1.15]	[0.96–1.31]

^a^Adjusted for cohort.

^b^Adjusted for cohort, father’s education, and school plans.

^c^Adjusted for cohort, father’s education, school plans, and education.

^d^Adjusted for cohort, father’s education, school plans, education, and physical demands.

^e^Adjusted for cohort, father’s education, school plans, education, psychological demands, and work control.

^f^Adjusted for cohort, father’s education, school plans, education, physical demands, psychological demands, and work control.

^g^Adjusted for cohort, father’s education, school plans, physical demands, psychological demands, and work control.

## Discussion

In this study, we found that preadolescent lower intelligence (at ages 12–13) was associated with all-cause disability pension, and also with disability pension due to mental disorder, musculoskeletal disorder and cardiovascular disease between ages 23 and 52, in both men and women. For men, there was also an association between lower intelligence and substance use disorder. The association between intelligence and disability pension granted for musculoskeletal disorder was stronger than the association between intelligence and disability pension granted for mental disorder. Among people with information on occupation in middle age, lower intelligence predicted musculoskeletal disorder. This association was partly mediated by adult work load, approximated by the job exposure matrices for physical and mental load, but remained increased in both men and women. Poor working environment is the main determinant of musculoskeletal disorder.

The HRs of all-cause disability pension, after controlling for cohort, showed a 1.20 per increase on the stanine scale for men, which and 1.13 on the scale for women, with both estimates statistically significant. The male HR is similar to that found in a previous study of age-18 intelligence and disability pension between ages 40 and 59 in a conscription cohort, HR = 1.26 per stanine step [18]. In the same cohort, low intelligence (defined as being in the stanine range 1–4 at age 18) was associated with disability pension from mental disorder between the ages 20 and 43 even after controlling for psychiatric diagnosis, psychological symptoms, and deviant behavior measured at conscription [17]. Also, our results are in line with those of a Norwegian conscription study, where the disability-pension HR decreased by about 7% per stanine increase in intelligence [16]. We know of no previous comparable studies of women.

The association found between intelligence and musculoskeletal disorder, was the only statistically significant multivariate association after excluding the first 15 years of follow-up. We found some reduction in the association after including attained level of education, which is similar to a result of the Norwegian conscript study, where one-third of the intelligence-disability pension association was found to be mediated through education [16]. In a British study, education fully mediated the associations of intelligence with anxiety and functional disability [31]. It is possible that intelligence also predicts disability pensions granted with other diagnoses, and that the associations would have been statistically significant with a larger sample.

We examined the role of working conditions by controlling for physical and mental demands, and also work control, in the participants’ jobs at ages 32–37, because these are factors that are associated with increased risk of disability pension due to musculoskeletal disorder, cardiovascular disease, and mental disorder [32]. We found, among those with occupational information in middle age, that work characteristics partially mediate the association between intelligence and musculoskeletal disorder, which indicates that at least some of the association is due to the selection of lower intelligence groups into more dangerous occupations. These findings are in line with those found in a cohort of 12 year-old boys and girls in Luxembourg, where the association between childhood intelligence and age-52 physical functional ability was fully mediated by education and adult socioeconomic position [33].

The crude association between intelligence and disability pension due to mental disorder, excluding substance use disorder, found in our study was HR 1.15 for men and HR 1.08 for women, for each point decrease on the stanine intelligence scale. This is in line with the finding of a Danish study of similar design (although with men only) in the same period, and using the same intelligence test, where every SD decrease in intelligence increased the HR of psychiatric hospitalization by 1.2 over 33 years of follow-up [34]. Larger studies of men with intelligence-test results collected at conscription have shown that intelligence is inversely associated with hospitalization due to most psychiatric diagnoses [35,36]. However these inpatients are characterized by injuries and high-severity disorders, while the Common Mental Disorders that give rise to the bulk of disability pension may be less associated with intelligence. Since we were able to adjust for adult physical and psychological work load, approximated in job exposure matrices, we provide additional understanding of how intelligence becomes related to future occupational exposure and disability-related exit from the labor market. Several mechanisms might underlie the association of intelligence with morbidity, including latent morbidity, coping and behavior, and selection into hazardous occupational positions. Hazardous occupational positions in adulthood, as well as educational success, and adult social circumstances are on the likely pathway, a chain of adverse events eventually leading to poor health and accompanying functional abilities. However, these adult circumstances might also be interpreted as indicators of a lower cognitive ability that would affect health irrespective of adult social and economic circumstances [37]. Using cause-specific disability pension provided one means for extending the knowledge of how and why intelligence predicts disorder and disability, although the etiology (e.g. work-related or not) of the disorders that accompany the disability pension is not known.

### Methodological considerations

The principal strength of this study is that is based on a large sample representative of the Swedish population, including women. No previous study has examined the association between intelligence and disability pension in women. A second strength is high baseline participation. With more than 90% participating children, the test results are likely to provide good estimates of the population’s intelligence. A third strength is the use of register data on diagnoses for disability pension, which were made by a physician. With the exception of one previous study based on Swedish men, where disability pension due to mental disorder was examined, studies of intelligence and disability pension have relied on non-specific disability-pension data.

Job control and physical strain at work were assessed using Job Exposure Matrices, where the average levels of exposure for specific occupational codes are used. A potential weakness of this procedure is that exposures are estimated rather than measured. The mean exposure level for an occupation might poorly represent what an actual individual experiences in his or her workplace, due simply to variance within occupations. However, self-reports of job exposures are less reliable because of self-report bias; that is, some underlying trait might produce both negative reporting and later disability pension. Although instruments like the Job Content Questionnaire (JCQ) strive to capture objective reports on rather than subjective evaluations of jobs [38], self-report bias is not ruled out. In a Finnish study, occupation-based JEM exposure was found to be a stronger determinant of disability pension than work-unit-based exposure (where the individual JCQ mean is subtracted from the work unit mean)[39]. If we had had more refined information, e.g., on working postures, manual handling, repetitive work and vibrations, even stronger support for an indirect rather than a direct association between intelligence and disability pension due to musculoskeletal disorder would probably have appeared. Another potential limitation is that the occupational codes in the census refer to the respondents’ occupation in a given week, and, although most individuals are occupationally stable in this regard, for some individuals, their occupation at a specific time point might not represent their long-term work exposure. However, comparisons between census occupations and survey reports on occupational history have shown good overall agreement, in particular with regard to the physical Job Exposure Index used in our study. [40,41]

Another caveat is that the validity of the ICD diagnoses involved in disability pension are largely unexamined. One study comparing registered sick leave due to musculoskeletal diagnoses (that are set by the same physicians as for disability pension) with relevant self-reported retrospective information (four years back) did, however, find a high level of agreement (89–97%) [42], and we see no reason why the diagnostic practice for disability pension should be different from the practice for sick leave.

### Conclusion

In this large population-based sample of Swedish men and women we found that preadolescent intelligence (at ages 12–13) was associated with disability pension between ages 20 and 61. Associations were found for disability pension granted for musculoskeletal disorder as well as for mental disorder and cardiovascular disease, in both men and women, but disability pension due to a musculoskeletal disorder was the only association that remained statistically significant after adjusting for education and work environment.
